# Fellows Hypophosphites

**Published:** 1892-02

**Authors:** W. Armstrong

**Affiliations:** 16, Brunswick Street, Fitzroy, Melbourne, Australia


FELLOW'S HYPOPHOSPHITE.—I have much pleasure in informing you that I have prescribed your Syrup of the Hypophosphites with most satisfactory results, principal y in cases of cerebral nervous exhaustion. I have found it specially beneficial in cases of tubercular insanity, where it appears to have exerted a calming effect upon the irritable condition of the brain, and so promoted sleep in cases in which the absence of the latter, had been conspicuous.

Yours truly, W. ARMSTRONG. ESQ., M. D., B. S.
16, Brunswick Street, Fitzroy, Melbourne, Australia.

